# Therapeutic class-specific signal detection of bradycardia associated with propranolol hydrochloride

**DOI:** 10.4103/0253-7613.56068

**Published:** 2009-08

**Authors:** Dhaval K. Gavali, Kala S. Kulkarni, Amal Kumar, Bhaswat S. Chakraborty

**Affiliations:** Department of Pharmacology, SPTM Shirpur Campus SVKM's NMIMS University, Mumbai, India; 1 Department of Clinical Research and Pharmacology, Cadila Pharmaceuticals Ltd, Ahmedabad, Gujarat, India

**Keywords:** Adverse drug reactions, canadian adverse drug reaction monitoring program, propranolol, signal detection

## Abstract

**Background:**

Propranolol hydrochloride, one of the most widely used β-blocker in the treatment of hypertension since 1960s, shows a number of serious and non-serious adverse events.

**Objective:**

Major objectives of this study were to extract the Canadian Adverse Drug Reaction Monitoring Program (CADRMP) database for possible toxic signal detection (SD) of propranolol hydrochloride, evaluate the frequency of the bradycardia associated with it in different stratified groups for a putative signal, and generate awareness in healthcare professionals regarding usefulness of SD.

**Materials and Methods:**

Appropriate statistical methods were used for adverse drug reaction (ADR) signal detection such as, proportional reporting ratio (PRR); reporting odds ratio (ROR); the Chi-square (χ^2^) statistic method; the 95% confidence interval (CI); the observed to expected ratio (O/E); and Du Mouchel method were used to calculate the possible signals. Significance of χ^2^ and other calculated statistics, e.g., PRR and ROR, was based on a composite criterion of regulatory guidelines and not on any particular statistical level of significance.

**Results:**

Calculated statistics by different methods were compared with the regulatory criteria of a statistic value ≥4.0 for χ^2^, and ≥3.0 for the rest for SD to be declared significant. The PRR statistic was found to be 2.5054; by the ROR method it was 2.5820; the χ^2^ statistic was 3.2598, whereas the lower and upper limits of 95% CI of PRR were found to be 0.0778 and 1.9104, respectively, by the O/E ratio was found to be 2.3978, and PRR with the help of Du Mouchel was found to be 2.3979. Thus, the bradycardia–propranolol signals calculated in this study were not significant.

**Conclusions:**

The therapeutic class specific signal of bradycardia associated with propranolol hydrochloride was not found potent enough to cause bradycardia. However, since the calculated statistics were very high albeit not significant, the possibility of bradycardia–propranolol pairing should still be analyzed from larger databases.

## Introduction

The WHO defines a toxic signal as: “Reported information on a possible causal relationship between an adverse event and a drug, the relationship being unknown or incompletely documented previously.”[1] Usually, more than a single report is required to generate a signal, depending upon the seriousness of the event and quality of the information.

Adverse drug reactions (ADRs) are thought to be the 4-6 largest cause of death in the USA and are estimated to cause 3-7% of all hospital admissions.[2] More than half of these ADRs are not recognized by the physicians on admission, and ADRs may be responsible for death of 15 of 1000 patients admitted.[3] There is a need of close link between the market authorization holder (MAH) and the pharmacovigilance (PV) system, allowing products to be authorized earlier under strict and clearly defined rules for post-authorization safety studies, thus offering hope to patients with currently unmet medical needs.[4]

The role of SD and PV do not end by establishing a drug–ADR pair only, prompt regulatory actions need to be taken to appropriately restrict or ban the drug. In the recent past, critical opinions have been aired about the sale of many drugs from some countries that have been banned in other countries.[5] All over the world, nimesulide has been withdrawn, but unfortunately it still continues to be sold in most countries.[6] Another drug that has been voluntarily withdrawn recently by the manufacturer (Merck) from the world market was Vioxx^®^ which was containing rofecoxib.[7]

The mortality rate associated with cardiovascular disease (CVD) concluded from International Mortality Data from World Health Statistics of World Health Organization (WHO) suggests that 9,53,110 deaths were caused by total CVD in the year 2000. Out of this 9,53,110 deaths, 48.5% died due to coronary heart disease (CHD), 16.7% died because of stroke, 4.5% died because of disease of arteries, and other CVD caused 2.4% and other heart diseases caused 27.8% of the total deaths although the percentage deaths caused by ADR is not specified.[8] With this high mortality rate there is an immense need of a perfect PV system, which can evaluate the risk benefit ratio of a drug used for CVD.

Hypertension is a common disorder, if not treated properly may lead to stroke, coronary thrombosis, and renal failure. Until about 1950, there was no effective treatment available to treat hypertension.[9] β-blocker therapy in the treatment of hypertension has been associated with improved cardiovascular outcomes. According to the 2003 JNC-VII guidelines for the treatment of hypertension, most patients with hypertension will require treatment with at least two antihypertensives. Compelling indications for a β-blocker include patients with heart failure, post-myocardial infarction, high coronary disease risk, or diabetes. While using a β-blocker, such as propranolol, sometimes severe bradycardia has been reported.[10] We undertook this project to study whether bradycardia indeed constitutes a toxic signal for propranolol.

## Materials and Methods

The three-fold methodology used in this study was as follows:

### Data extraction from public database

Extraction of relevant data from CADRMP database was carried out as follows. First, the Health Canada website (http://www.hc-sc.gc.ca/index-eng.php) was accessed; then the following sections were serially accessed: Drug and health products and MedEffect Canada Adverse Reactions. Finally, in the section of Canada Vigilance Program, the CADRMP online database was extracted.

### Procedure followed for signal detection in this study

**Figure 1:**
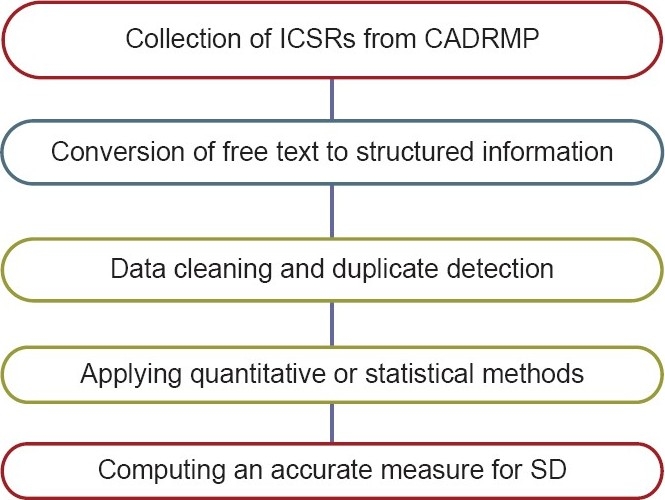
Procedure followed for Signal Detection by statistical and quantitative methods. The sub-database created here for computing a signal consists of only Iindividual case safety Reports reported for particular therapeutic class of anti-hypertensive drugs. While calculating an actual signal, duplicate reports were eliminated

The CADRMP is the vigilance program database of Health Canada. The Individual Case Safety Reports (ICSRs) in this database were collected by forwarding a request to health Canada, and the remaining ICSRs were collected from the official website of health Canada. The free text collected from the CADRMP was converted into a structured format, and finally statistical methods were applied to calculate an actual measure of signals. Therapeutic class-specific SD calculations were then carried out as shown in Figure 1.

Various methods, appropriate for analyzing a large number of reports, were used for SD calculations. These methods of calculations were selected following a systematic literature review.[11–12] An attempt was made to maintain the originality of data collected from CADRMP database while computing actual SD.

### Signal detection calculations

Disproportionality: Disproportionality or signals of disproportionate reporting are the frequency or relative frequency of a particular drug–event pair. When the statistics from different calculations, PRR, ROR, χ^2^, the 95% CI, the O/E ratio, and Du-Mouchel method did exceed a certain value (for χ^2^ to be ≥ 4.0 and, for the rest to be ≥ 3.0) then the signal would be considered significant.[12]

The Proportional Reporting Ratio: As shown in Table 1, a 2*2 contingency Table was prepared to capture the incidences of bradycardia (targeted event Y) and all other events for the targeted drug X, i.e., propranolol, and all other drugs in the database to calculate PRR.[11]

**Table 1:** 2*2 Contingency table for the computation of proportional reporting ratio

	*Targeted event Y*	*All other events*	*Total*
Targeted drug X	A	B	A+B
All other drugs	C	D	C+D
Total	A+C	B+D	A+B+C+D

In this Table the elements counted A, B, C, and D were ICSRs available in the CADRMP database. Thus, a given ICSR may contribute to only one of the cells of the Table event, if the individual case refers to multiple medicinal products or multiple adverse events

The PRR was calculated as follows:

(1) $$\text{PPR}=\frac{\text{A}/\left(\text{A}+\text{B}\right)}{\text{C}/\left(\text{C}+\text{D}\right)}$$

The Reporting Odds Ratio: The procedure followed to calculate ROR was similar to that of the PRR method.[11] The same contingency Table [Table 1], prepared for PRR, was also followed in the case of ROR calculations.

The ROR was calculated as follows:

(2) $$\text{ROR}=\frac{\text{A}/\text{B}}{\text{C}/\text{D}}$$

The Chi-square (χ^2^) statistic: The Chi-square statistic[12] was applied to test the independence of categorical variables. χ^2^ was used as an alternative measure of heterogeneity in the contingency Table built with the medicinal product X and the adverse event Y.

(3) $${\text{χ}}^{2}=\frac{{\left(\text{Observed}-\text{Expected}\right)}^{2}}{\text{Expected}}$$

#### The 95% confidence interval of the PRR:

The standard error of the natural logarithm of the PRR was estimated based on the following formula:

(4) $$\text{SE}=\sqrt{1/\text{A}+1/\text{C}-1/\left(\text{A}+\text{B}\right)-1/\left(\text{C}+\text{D}\right)}$$

The 95% CI for ln (PRR) was then estimated as ln (PRR) = 1.96SE, and its exponential was taken.[12]

(5) Lower and upper limits of 95% CI for PRR=[PRR/exp(1.96SE), PRR×exp(1.96SE)]

#### The observed-to-expected (O/E) ratio:

The was O/E[11] calculated as follows:

(6) $$\text{OE}=\frac{\text{A}/\left(\text{A}+\text{B}\right)}{\left(\text{A}+\text{C}\right)/\left(\text{A}+\text{B}+\text{C}+\text{D}\right)}$$

#### Du Mouchel Method:

This method was based on 2*2 contingency Table values as well as the ratio of values of A and expected A was taken into consideration for calculations.

(7) $$\text{PRR}=\frac{\text{A}/\left(\text{A}+\text{B}\right)}{\left(\text{A}+\text{C}\right)/\text{N}}$$

(8) $$\text{E}\left(\text{A}\right)=\frac{\left(\text{A}+\text{B}\right)\left(\text{A}+\text{C}\right)}{\text{N}}$$

(9) $$\text{PRR}=\frac{\text{A}}{\text{E}\left(\text{A}\right)}$$

The statistical significance of PRR, ROR, χ^2^, O/E ratio, and Du-Mouchel statistics was based on regulatory guidelines[12] as mentioned above.

## Results

The details of calculated statistics are presented as follows:

### Canadian adverse drug reaction monitoring program

The Canadian Adverse Drug Reaction Monitoring Program (CADRMP) is the Health Canada's post-market surveillance program which collects and assesses suspected adverse reaction reports for Canadian marketed health products such as pharmaceuticals, biologics (including fractionated blood products, as well as therapeutic and diagnostic vaccines), natural health products, and radiopharmaceuticals.[13]

### Characteristics of system organ class

The occurrence of ADRs in primary system organ class (SOC) affected causing 20% metabolism and nutrition disorders, 60% nervous system disorders, 20% eye disorders, 100% cardiac disorders, 60% vascular disorders, 20% respiratory, thoracic mediastinal disorders, 20% gastrointestinal disorders, 20% general disorders and administration site conditions, 40% investigations, 30% injury, 40% poisoning and procedural complications. The data received clearly indicate that 100% reports met the seriousness criteria – involving one death, one life-threatening situation and two reports of hospitalization required (out of total four reports). The two females and two males, respectively, reported bradycardia associated with the propranolol hydrochloride out of four reports.

### Essential data for Signal detection

The following data were extracted from CADRMP database:

- Total ICSRs included in database = 977
- Bradycardia associated with propranolol hydrochloride = 04
- Other ADRs reported with propranolol hydrochloride = 82
- Bradycardia associated with other than propranolol hydrochloride = 52
- Other ADRs associated with other than propranolol hydrochloride = 2749
- These data were organized into contingency table [Table 2].

**Table 2:** Data obtained from canadian adverse drug reaction monitoring program database to calculate signal detection

	*Bradycardia*	*Not bradycardia*
Propranolol HCL	4	82
Not propranolol HCL	52	2749

### PRR

(1) $$\begin{array}{cc} \text{PRR} & =\frac{\text{A}/\left(\text{A}+\text{B}\right)}{\text{C}/\left(\text{C}+\text{D}\right)}\\ & =\frac{4/\left(4+82\right)}{52/\left(52+2749\right)}\\ ∴\text{PRR} & =2.5054 \end{array}$$

The PRR calculated from Table 2 was 2.5054.

### ROR

(2) $$\begin{array}{cc} \text{ROR} & =\frac{\text{A}/\text{B}}{\text{C}/\text{D}}\\ ∴\text{ROR} & =\frac{4/82}{52/2749}\\ \text{ROR} & =2.5820 \end{array}$$

The ROR calculated from Table 2 was 2.5820.

(3) $$\begin{array}{ll} {\text{χ}}^{2}: & \\ {\text{χ}}^{2} & =\frac{{\left(\text{Observed}-\text{Expected}\right)}^{2}}{\text{Expected}}\\ & =\frac{{\left(4-1.6681\right)}^{2}}{1.6681}\\ {\text{χ}}^{2} & =3.2598 \end{array}$$

The χ^2^ statistics was calculated from the data organized in Table 3 was 3.2598.

**Table 3:** Data obtained from canadian adverse drug reaction monitoring program database to calculate signal detection

	*Bradycardia*	*Not bradycardia*	*Total*
Propranolol HCl	04	82	86
Not propranolol HCl	52	2749	2801
Total	56	2831	2887

### 95% CI for PRR

(4) $$\begin{array}{cc} \text{SE} & =\sqrt{1/\text{A}+1/\text{C}-1/\left(\text{A}+\text{B}\right)-1/\left(\text{C}+\text{D}\right)}\\ \text{SE} & =\sqrt{1/4+1/52-1/86-1/2801}\\ \text{SE} & =0.5079 \end{array}$$

(5) $$\begin{array}{l} 95\%\text{ CI for PRR}=\text{In}\left(\text{PRR}\right)\pm 1.96\text{ SE}\\ =\text{In }\left(2.50\right)\pm 1.96\left(0.5079\right)\\ =0.9186\pm 0.9954 \end{array}$$

Lower and upper limits of 95% CI for PRR = 0.0778 and 1.9104

The SE calculated for ln(PRR) was 0.5079. Using this value, the lower and Uuper limits of 95% CI of PRR were found to be 0.0778 and 1.9104, respectively.

### O/E ratio

(6) $$\begin{array}{cc} \text{OE} & =\frac{\text{A}/\left(\text{A}+\text{B}\right)}{\left(\text{A}+\text{C}\right)/\left(\text{A}+\text{B}+\text{C}+\text{D}\right)}\\ & =\frac{4/\left(4+82\right)}{\left(4+52\right)/\left(4+82+52+2749\right)}\\ \text{OE} & =2.3978 \end{array}$$

The O/E ratio for bradycardia-propranolol pair was found to be 2.3978.

### Du-Mouchel parameters

(7) $$\text{PRR}=\frac{\text{A}/\left(\text{A}+\text{B}\right)}{\left(\text{A}+\text{C}\right)/\text{N}}$$

where

A = 4, targeted adverse event Y caused by particular drug X in database;

B = 82, all other adverse events caused by drug X except Y in database;

C = 52, targeted adverse event Y caused by all other drugs except X in database;

D = 2749, all other adverse events caused by all other drugs except drug X in database;

N = 2887, total sum of each cell of Table 2.

(8) $$\begin{array}{cc} \text{PRR} & =\frac{4/86}{56/2887}\\ \text{PRR} & =2.3978\\ \text{E}\left(\text{a}\right) & =\frac{\left(\text{A}+\text{B}\right)\left(\text{A}+\text{C}\right)}{\text{N}} \end{array}$$

where E (a) = expected value of A i.e. Bradycardia associated with propranolol hydrochloride

(9) $$\begin{array}{cc} & =\frac{\left(86\right)\left(56\right)}{2887}\\ \text{E}\left(\text{a}\right) & =1.6681\\ \text{PRR} & =\frac{\text{A}}{\text{E}\left(\text{a}\right)}\\ & =\frac{4}{1.6681}\\ \text{PRR} & =2.3979 \end{array}$$

The E(a) was calculated to be 1.6681, whereas the PRR value by Du-Mouchel was found to be 2.3979.

### Data by gender and age group

The data received from CADRMP database was converted into a structured format and then it was stratified into two groups on the basis of gender. The bradycardia associated with propranolol hydrochloride was found to be more prominent in male as high as 52% as compared to 48% in female. In another method, data were stratified into three different groups on the basis of age: Group A: 0-18 years, Group B: 19-60 years, and Group C: 61-100 years showed 5%, 43%, and 52% bradycardia, respectively, associated with propranolol hydrochloride.

### Significance of calculated Signal detection parameters

The PRR was calculated by incorporating all the values of CADRMP database in Table 2 and applying Equation 1. It should be noted that if C = 0 in Table 2, then PRR cannot be calculated. The calculated PRR was found to be 2.5054 which was <3.0, and hence, the bradycardia incidences could not be called a potential toxic signal.

The ROR (Equation 2) was found to be 2.5820. Although this value is high (much more then 1.0), it could not be called a signal by regulatory criteria (≥3.0). For a signal, when a PRR was displayed with χ^2^ statistics, the PRR should be ≥2 and the x^2^ should be ≥4 by the regulatory criteria.[12] The individual number of cases should be more than three. The χ^2^ was found to be 3.2598, therefore fall short of being a signal.

Similarly, the results of 95% CI and Du-Mouchel parameters were not significant as signals. Summary results of all statistical and quantitative calculations are provided in Table 4.

**Table 4:** Summary results of all statistical and quantitative calculations

*SD parameter*	*Results*
PRR	2.5054 not significant
ROR	2.5820 not significant
Chi-square test	3.2598 not significant
95% confidence interval	0.0778–1.9104 not significant
Observed to expected ratio	2.3978 not significant
Du-Mouchel method	2.3979 not significant

## Discussion

The data received from “line listing” request to CADRMP indicate that in 10 years of time (01/01/1998–01/01/2008), only four ICSRs of bradycardia associated with the propranolol hydrochloride were reported.

For therapeutic class-specific SD of bradycardia associated with the propranolol hydrochloride the ICSRs were collected from CADRMP online database.

Extracting all the necessary information and the background database is a very tedious job. All necessary data for SD from each individual ICSR were collected and placed in an appropriate format. The structured format for SD includes the Serial number, Report ID number, Date received, Patient information, ADR specified like bradycardia or other than bradycardia, and details about suspected drugs.

The essential data (see Essential Data for SD in Results and Data Overview section) were cleaned up and duplicate reports were eliminated. The data, which were converted into structured information format, were included in Table 1, to get the values of A, B, C, and D. The confirmation about correct values received was also checked by the sum total of all cells (N).

The PRR and other calculations involve comparisons of reporting relationship for a specific medicinal product X and a targeted adverse event Y with all other medicinal products in database. When we considered the therapeutic class of anti-hypertensive drugs as the background data, it should be noted that our results might differ slightly from the signal produced by the propranolol hydrochloride–bradycardia pair reported using a whole database.

There was no gender difference in the incidences of bradycardia; however, the geriatric patients above 61 years of age definitely showed higher incidences of bradycardia.

The initial decision on whether a drug–event pair should be further investigated was based on composite regulatory standards[12] applied to the estimates of the PRR and other statistics. There was no pure “significance level” applied to all calculations of SD.

The value of the PRR and consequently the signal detected with this method depend on the data in the database on which the PRR was computed. Therefore, the PRR interpretation should take the following elements into account:

- The type of medicinal products included in the database.
- The medicinal terminologies that have been used in the reports populating the database.
- The coding practices.
- The sources of ICSRs.The absence of signal detected in this database does not necessarily exclude the possibility of an association between the medicinal product X (propranalol hydrochloride) and the adverse event Y (bradycardia) and therefore further investigations are required in other databases.

The PRR may be refined by using similar techniques to other SD like combining multiple medicinal products and or adverse events, stratification by age and sex of the patient.

The novelty of this work includes a method that enables SD calculations in spite of the limitations of extracting whole database. The new idea generated in this paper shows a way of calculating SD parameters within a therapeutic class when a large number of reports are available within that class. The same ideas can be implemented for SD in specific populations and also for stratified data on the basis of gender, age, and year.

The SD is a vital and essential part of drug use and surveillance information. With the help of SD, the authorities and practitioners can easily minimize the number of unwanted drug withdrawals. The class-specific SD procedures described in this paper, owing to the analysis of a large number of reports, will eventually facilitate the clinical safe use of the drug and promote patient care.
